# Experimental formation of carbonates from perchlorate and sulphate brines: Implications for Jezero crater, Mars

**DOI:** 10.1371/journal.pone.0312495

**Published:** 2024-12-05

**Authors:** Elizabeth Escamilla-Roa, Javier Martin-Torres, María-Paz Zorzano

**Affiliations:** 1 Instituto Andaluz de Ciencias de la Tierra (CSIC-UGR), Granada, Spain; 2 Department of Planetary Sciences, School of Geosciences, University of Aberdeen, Aberdeen, United Kingdom; 3 Centro de Astrobiología (CAB), CSIC-INTA, Madrid, Spain; Saveetha Institute of Medical and Technical Sciences: Saveetha University, INDIA

## Abstract

Extensive carbonate precipitation has occurred on Mars. To gain insight into the carbonation mechanisms and formation processes under ancient Martian aqueous conditions, we examine the precipitation of carbonates resulting from atmospheric carbon fixation, focusing on interactions between various brines and silicate and perchlorate solutions in alkaline environments. The micro-scale morphology and composition of the resulting precipitates are analysed using ESEM micrographs, EDX chemical compositional analysis, X-ray diffraction, and micro-Raman spectroscopy. Our findings indicate a significant atmospheric carbonation process involving chlorate and sulphate brines reacting with alkaline perchlorate solutions, leading to the precipitation of calcium carbonate polymorphs, including vaterite, aragonite, and calcite, as well as other carbonates like siderite (iron carbonate) and zaratite (nickel carbonate). Some precipitates exhibit biomorphic structures (such as globular spherical aggregates, fine branched tubes, and flower-like morphologies) that should not be mistaken for fossils. These experiments demonstrate that various precipitates can form simultaneously in a single reaction vessel while being exposed to different micro-scale pH conditions. We propose that systematic laboratory studies of such precipitate reactions should be conducted in preparation for the analysis of the Mars Sample Return collection on Earth, aiding in the interpretation of carbonate presence in natural brine-rock carbonation processes under Martian conditions while also helping to distinguish potential biosignatures from purely geochemical processes.

## Introduction

Within approximately ten years from now, the Mars Sample Return campaign will bring to Earth a collection of samples that are now being acquired by the Perseverance rover from Jezero Crater, Mars [1–5]. Carbonate detection from orbit on Mars is rare, but one of the unique features of the Jezero crater region is the existence of a vast carbonate-rich region that is distinguishable from orbit within the inflow channel to the ancient lake [6–10]. Zastrow and Glotch have detected, using visible/near-infrared hyperspectral imagery over Jezero, carbonate abundances up to ∼35% and identified three distinct units containing different carbonate phases, which points, presumably, to multiple periods of carbonate formation under varying aqueous conditions.

The remote detection of carbonates in association with a sedimentary environment that once hosted an intense water cycle was one of the reasons why the Jezero crater was chosen as the target of the in-situ investigation of Perseverance. This environment was also selected because of its interest in acquiring a diverse collection of samples of high astrobiological value that will be brought back to Earth for further analysis. On Mars, carbonation processes could involve the following steps: (i) adsorption of CO_2_ onto mineral surfaces; (ii) reaction of CO_2_ with water vapor to form carbonic acid (H_2_CO_3_); (iii) chemical weathering of minerals by carbonic acid, leading to the release of metal ions (e.g., Ca^2+^, Mg^2+^) and formation of carbonate minerals (e.g., calcium carbonate, magnesium carbonate); and (iv) precipitation of carbonates in pore spaces or fractures within rocks. As carbonates are formed when atmospheric carbon dioxide (CO₂) reacts with liquid water, a detailed analysis of the exact composition and distribution of carbonates within a rock can inform of the paleo-depositional environment. For instance, carbonates on Earth form in the shallow areas of freshwater or alkaline lakes, but they can also form through mineral carbonation when silicate minerals react with CO₂ and are converted to carbonate. Furthermore, carbonates are also interesting as they may preserve biosignatures [11]. Some potential biomarkers that could be found in Martian carbonates include (i) organic compounds: carbonates could contain organic molecules such as amino acids, lipids, or sugars, which could be remnants of past biological activity [12]; (ii) isotopic signatures: isotopic ratios of carbon, oxygen, and other elements within carbonates could provide clues about biological processes or environmental conditions during carbonate formation [13]; (iii) microbial fossils: microorganisms or their remains could be preserved within carbonate minerals as microfossils or biosignatures, providing direct evidence of past life [14]; or (iv) biosignature minerals: or some minerals associated with microbial metabolisms, such as certain iron minerals produced by iron-reducing bacteria, could potentially be found in association with carbonates as indicators of past life, i.e., carbonates may be produced as a by-product of microorganisms’ activity [15], so, in some cases, carbonates may also be considered themselves, under certain conditions, as a potential biomarker. When writing this article, Perseverance explores a band of carbonates along the inner edge of Jezero’s western crater rim. A possible hypothesis for the deposition of this carbonate-rich region suggests that more than 3 billion years ago, an alkaline lake in Jezero Crater might have reached its shores, depositing the carbonate layer [8].

The in-situ analysis of the rocks of Jezero crater by the instrumentation onboard Perseverance rover has provided new evidence of various degrees of aqueous activity that has altered the rocks at the base of Jezero crater in the form of water-soluble salts (chlorides and perchlorates), carbonate minerals, sulfates, iron oxides, and iron silicates [5]. The payload of Perseverance is unique and has implemented a detailed microscopic compositional and spatial distribution analysis, providing new insight into the processes that have affected a Martian environment. In particular, the analysis of the abraded patches of rocks of Séítah suggests the existence of olivine cumulate grains that were altered by fluids far from chemical equilibrium at low temperatures. These rocks, of igneous origin, were partially dissolved and filled by finely crystalline or amorphous secondary silicate, carbonate, sulfate, and chloride/oxychloride minerals. The analysis done on Mars by the instrument payload of Perseverance suggests that this region is an olivine and pyroxene-bearing unit containing minor amounts (<10%) of Mg-Fe carbonates, together with CaSO_4_ and MgSO_4_*nH_2_O as well as perchlorates [5].

At planetary scales, carbonates have also been detected in Martian meteorites [16] and in situ by different rovers such as Spirit, which identified outcrops of carbonate minerals in Gusev Crater [17]. Sulfates have also been found before on Mars in situ. In particular, at the landing site of the Opportunity rover, hydrated sulfates such as bassanite (CaSO_4_.1/2H_2_O), kieserite (MgSO_4_.H_2_O), [18] and jarosite (KFe^+3^(SO_4_)_2_(OH)_6_ were found. Curiosity rover also found magnesium sulfate at the Gale crater [19]. Although carbonate formation processes have happened widely on Mars, the carbonation mechanisms under Martian conditions are still poorly understood [20].

On the other hand, it is known from laboratory experiments that carbonation by atmospheric carbon fixation can occur in alkaline-basic fluid reactions where carbonates precipitate. In this kind of abiotic chemical reaction, microstructures rich in carbonates are formed, which sometimes mimic bioforms that should not be misinterpreted as true biomarkers. In preparation for the future geochemical analysis of the Mars sample return collection on Earth, we simulate the abiotic production of carbonates within some reaction-precipitation processes associated with some of the aqueous-alteration phases that these rocks may have been exposed to. We have investigated the precipitates at the microscale level to illustrate the different structures and compositional distributions that may appear in reaction precipitation processes produced by different brines in reaction with basic solutions to demonstrate the potential complementary use of different instrumentation.

### Carbonation precipitation reactions

Since the XVII^th^ century, it is well known that self-assembled tubular structures, also known as chemical gardens, can form biomimetic structures during reaction-precipitation processes when metallic salts are immersed in a basic solution forming a semipermeable membrane [21]. The self-assembled tri-dimensional structures, with different sizes and shapes, are a product of selective fluid transport through the semipermeable membrane due to a difference in osmotic pressure. The rupture of this membrane produces the ejection of the internal dissolution once in contact with the external media, which triggers the precipitation of some new products. This process happens naturally on Earth and is essential in forming mineral deposits in caverns and hydrothermal vents on the ocean floor [22]. In the case of samples from Mars, the detection of tubular structures can also be considered a plausible marker for the ancient presence of water [23, 24]. Chemical gardens have been used to study the crystal growth of several salts using sodium silicates [22, 25, 26]. Recently, this crystalline growth method has been applied to simulate the formation of carbonates under Martian conditions, *i*.*e*. considering a rich CO_2_ atmosphere and sodium silicate dissolution [24]. Here, we investigate carbonation processes experimentally with perchlorates and sulphate brines to emulate some of the aqueous alteration phases that may have existed on Mars, particularly at the Jezero crater.

## Materials and methods

### Laboratory experiments

Table 1 summarises the conditions of our experiments. They were performed at 1 atm, 20°C and under terrestrial ambient conditions (with 400 ppm of CO_2_), using different growth methods. Self-assembling structures were formed in the solid-liquid phase from an alkaline sodium perchlorate solution (NaClO_4_). Sodium perchlorate was freshly generated with HClO_4_ and NaOH. In the solid phase, different salts were considered, such as calcium chloride (CaCl_2_, anhydrous) and sulphate salts such as iron and hydrated nickel sulphates FeSO_4_.7H_2_O and NiSO_4_.7H_2_O. To include chlorate and silicates, we considered a mixture of potassium chlorate (KClO_3_) and sodium silicate (Na_2_SiO_3_.5H_2_O) dissolutions. All reagents had analytical purity and were purchased from Sigma Aldrich ©.

**Table 1:** Summary of the experimental conditions: Crystal seed, crystal growth method, dissolution type and concentration [M] and experiment.

Experimental reactants	Growth method	Crystal seed			pH control	Experiments
		CaCl_2_ FeSO_4_.7W^a^ NiSO_4_.7W^a^				
NaClO_4_	grains	2M	2M	2M	11.90	A
		3M	3M	3M	12.00	B
	pellets	2M	2M	2M	11.90	C
		3M	3M	3M	12.00	D
KClO_3_+ (Na_2_SiO_3_.5H_2_O)	grains	0.6	0.6	--	9.02	E

^a^7W = 7H_2_O

To investigate the role of the size of the seed (and the interface with the solution) and the possible role of a homogenised mixture of seeds, two types of seeds were tested to induce crystalline growth: untreated grain and prepared pellets.

#### a) The solid phase seed as an untreated grain

In the untreated grain method, several salt grains (as procured) were introduced into a small reactor with two dissolution types: 1) with 3 and 7 ml of sodium perchlorate solution at different concentrations (2 or 3 M, experiments A and B) where the pH varies between 11.9 and 12.00 (see Table 1); and 2) given that there is experimental and theoretical evidence supporting that substrate can be formed with chlorate and silicate on Mars’s surface [27, 28], we consider a substrate that is formed from a basic dissolution (0.6 M) and pH of 9.02 with a mixture of sodium silicate and potassium chlorate in which CaCl_2_ and FeSO_4_.7H_2_O salts are presents (experiment E).

#### b) The solid phase as a pellet

In the pellet method, 0.069 mg of hydrated NiSO_4_, 0.069 mg of hydrated FeSO_4_ and 0.072 mg of CaCl_2_ were homogenised with an agate mortar and pressed into cylindrical pellets of 13 mm diameter and 1 mm of thickness using a cell at a pressure of 10 bar over 10 min. The pellets were introduced into a reactor of 15 ml. At that moment, 10 ml of basic solution at a high concentration of sodium perchlorate (2 and 3 M, experiments C and D, respectively, with pH 11.9 and 12) were poured into each reactor.

In both methods, the seed or pellet was left at ambient conditions in the basic solution for 24 hours to allow all precipitation processes to finish. Then, the precipitates were washed twice with Mili-Q water to remove the basic solution and dried at room temperature to be characterised by different analytical techniques.

### Analysis techniques

We have used several analytical methods to analyse our results: 1) micrographs of the samples using a Jeol JSM-IT300 Environmental Scanning Electron Microscope (ESEM); 2) chemical analysis of the micromorphology observed by ESEM *in situ* in the microscope by using EDX (Electron detector of X-ray absorption) analysis; 3) PANalytical X’Pert PRO diffractometry for the identification of the crystallographic phases by XPOWDER© X-ray diffraction (XRD). Diffractograms were analysed using the XPOWDER© code [29]; 4) micro-Raman spectroscopy analyses with a JASCO NRS-5100 spectrometer connected to a microscope using a visible/near-infrared (VIS-NIR) laser of 785 nm and 30 mW, with five cycles of 20 s of acquisition time.

## Results and discussions

### 1. Global morphology analysis

#### a) Experiments A and B: NaClO_4_ and untreated grain crystalline growth method

**Fig 1:**
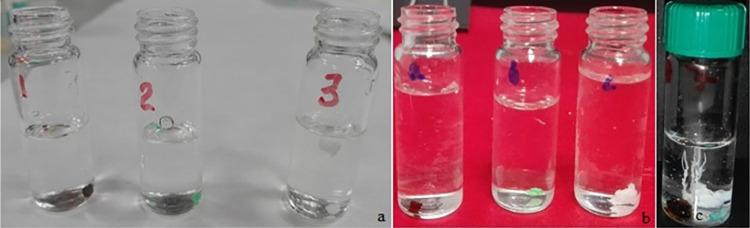
Perchlorate gardens in the solid-liquid phase: (a) Experiment B, FeSO_4_.7H_2_O, NiSO_4_.7H_2_O and CaCl_2_ salts at 3M of sodium perchlorate (1, 2 and 3 labels respectively) (b) Pellet method and 2M concentration (Experiment C), with the same order as experiment B and (c) Experiment E. White, brown and green correspond to the structures of Ca^2+^, Fe^2+^ and Ni^2+^ salts respectively.

**Fig 2:**
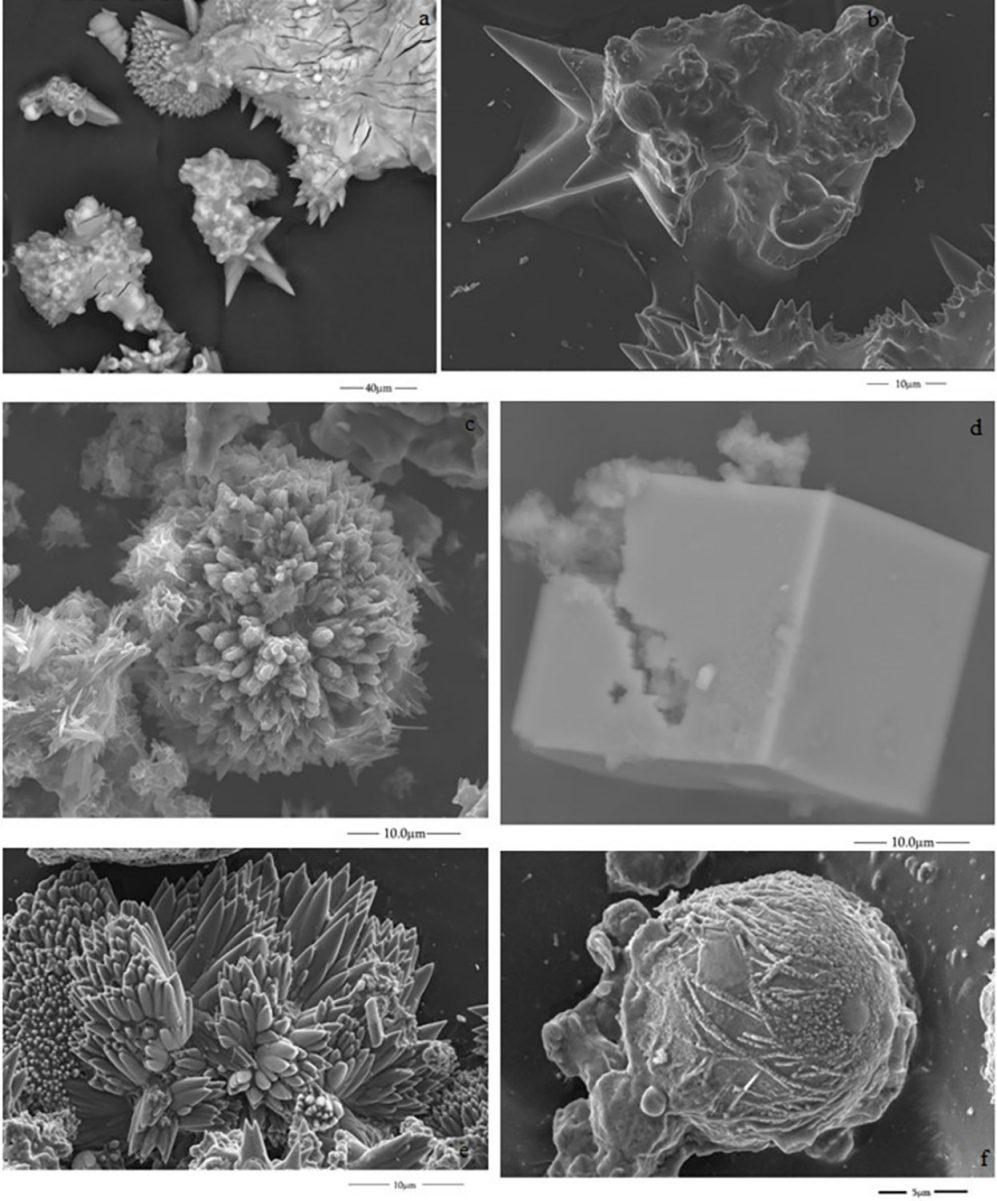
ESEM Micrographs of the inner surface of the tube of structures formed from the untreated grain method with CaCl_2_: (a and b) and sodium perchlorate dissolution 2M (experiment A) and (from c to f) 3M concentration (experiment B).

**Fig 3:**
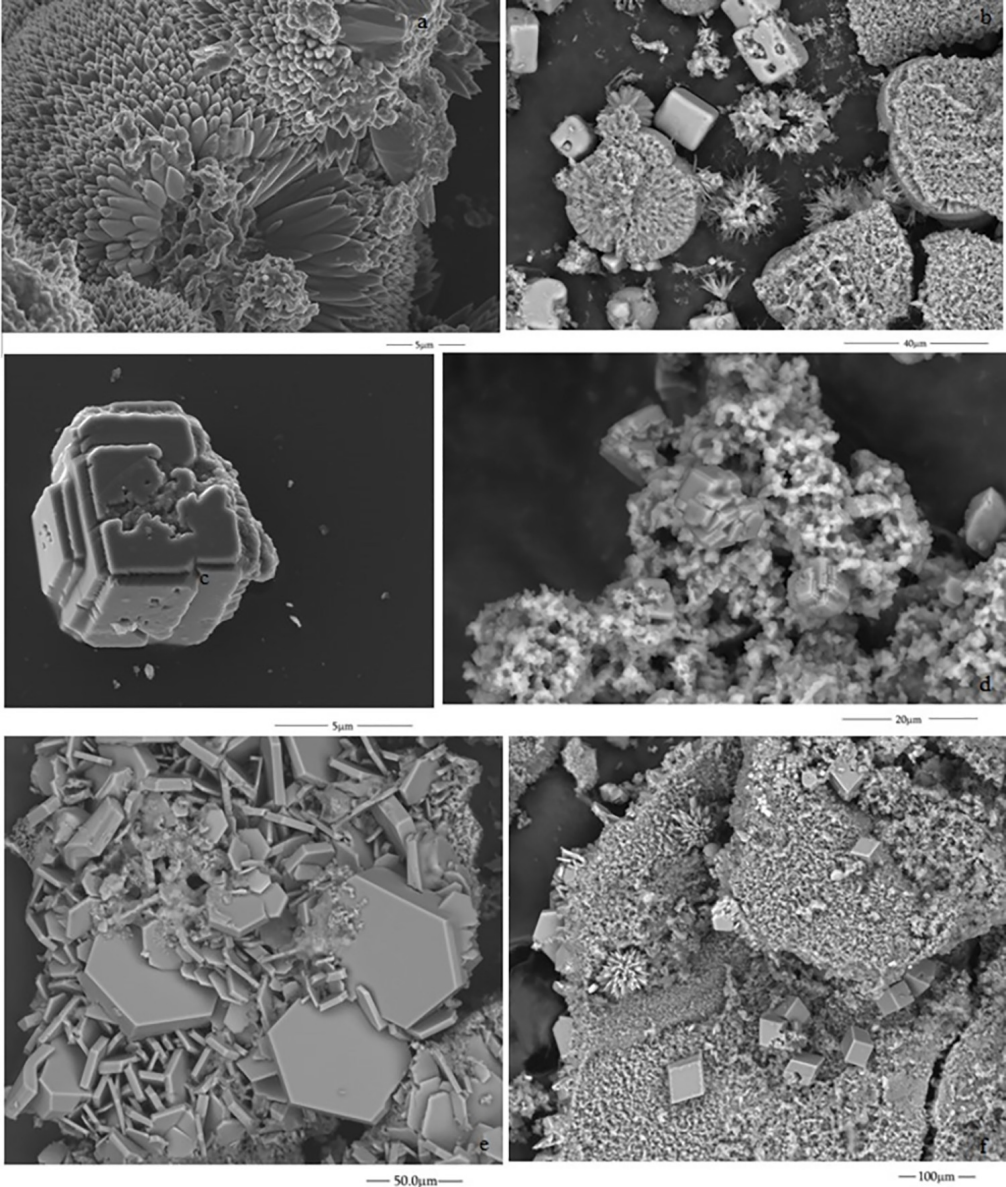
ESEM micrographs of the inner surfaces of the tubular structures obtained with CaCl_2_: (a and b) 2M (experiment C) and (c and d) 3M (experiment D) in pellet method and (f and g) with sodium silicate and potassium chlorate mixture (experiment E).

The self-assembling structures formed in the untreated grain method from sodium perchlorate at 2M (experiment A) and 3M (experiment B) and CaCl_2_ has formed a spherical aggregate (Fig 1A–3). Also, with similar conditions but using sulphate salts, we did not see the typical growth of tubular chemical gardens, only spherical aggregates (Fig 1A labels 1 and 2). The seeds swelled, acquiring globular forms, and, in some cases, some of these spheres were detached and grown further on the surface (Fig 1A–3). This behaviour was observed in our previous experiments related with self-assembled structures formed from sodium silicate dissolution and a dense CO_2_ atmosphere [24].

#### b) Experiment C and D: NaClO_4_ and pellet crystalline growth method

For both concentrations used in experiments C and D, and in the same way as in the untreated grain method, the self-assembling structures from CaCl_2_ and sodium perchlorate formed a small and fine white tube (Fig 1B and 1C). However, in both experiments, the hydrated NiSO_4_ and FeSO_4_ also formed spherical aggregates (Fig 1B labels a and b).

#### c) Experiment E: Sodium silicate and potassium chlorate solution

The self-assembling structures formed in the untreated grain method with a mixture of potassium chlorate 0.6 M and basic dissolution of sodium silicate with CaCl_2_ and sulphate salts have the same behaviour as the previous experiments, *i*.*e*., the sulphate salts also formed spherical aggregates. In contrast, the calcium chloride formed numerous fine, white, branched tubes, see Fig 1C. Like experiments A and B, this crystal growth was reproduced in structures formed from the sodium silicate dissolution [24].

### 2. Characterisation

#### 2.1 Crystal growth of self-assembling tubular structures with calcium chloride salt

We used the environmental scanning electron microscopy (ESEM) analytical method to determine the type of crystal and its distribution and morphology within the self-assembled structures. The micrographs suggest the formation of several polymorphs of calcium carbonate, such as vaterite, aragonite and calcite.

- Experiment A: We found a characteristic star morphology that could be assigned to a typical aragonite particle (Fig 2A and 2B). This morphology has been reported in our recent study of calcium carbonate growth with a rich, dense CO_2_ atmosphere [24].
- Experiment B:
  We observe needle-shaped aggregates forming flower-like morphologies. These structures formed at 3M dissolutions have also been reported as aragonite polymorphs (Fig 2C) [30]. In addition, flower-like and cubic crystals were formed in the inner surface of the tube (Fig 2D and 2E) that are like previously reported calcite crystals [31–33]. Previous investigations indicated that these peculiar morphologies result from the pH increase between 11–12, where there are transformations from vaterite to calcite, and the flower morphology is a result of the re-growth processes of calcite particles [31]. In other cases, the flowers can also correspond to vaterite [32]. Our experiments were run at high pH values, so our results are consistent with these previous investigations. The spherical micromorphology is shown in Fig 2F, which reminds us of a microfossil. These forms were reported as calcite before [33].
- Experiments C and D
  In the pellet method with perchlorate 2M dissolution, the structures formed in the inner parts are flowers, globular spherical aggregates, and cubic crystals (Fig 3A and 3B). Cubic shapes prevail for the experiments at 3M concentration (Fig 3C and 3D).
- Experiment E
  In the same way, rhombohedral, flower-like and cubic crystals (Fig 3E and 3F) were formed from the dissolution of sodium silicate and potassium chlorate mixture, as mentioned above.

**Fig 4:**
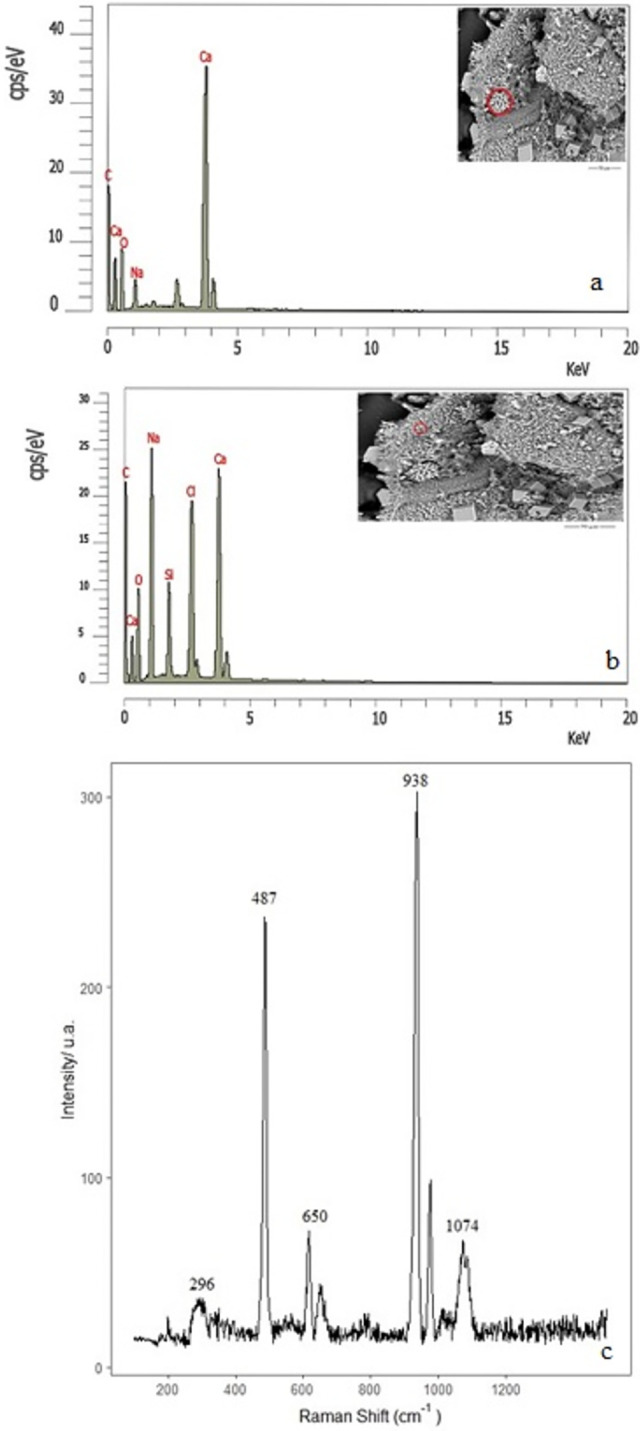
Chemical microanalysis of structures formed from CaCl_2_ and a mixture of potassium chlorate and sodium silicate and micro-Raman spectrum (experiment E).

The ESEM chemical microanalysis of the structures formed from the mixture dissolution (experiment E) indicated that the flower’s petals and the cubic forms could correspond to any CaCO_3_ polymorph (Fig 4A). On the contrary, the granular surface is composed of a higher proportion of calcium, oxygen, chlorine, and sodium, indicating the presence of calcium oxides-hydroxides along with CaSiO_3_ and the rest of the reactant (Fig 4B). We have made a micro-Raman analysis (Fig 4C) to confirm our hypothesis about the growth of calcium carbonate polymorphs in the interior of the surface. The spectrum shows the characteristic bands of vaterite at 1074 and 300 cm^-1^ and probable calcite and aragonite at 1080 cm^-1^, which could be masked in the broad peak at 1074 cm^-1^ [34–36]. This result agrees well with the flower-like vaterite crystal aggregates previously reported [32]. Also, another defined peak at 938 cm^-1^ could correspond to the characteristic signature of sodium perchlorate at 933 cm^-1^. The presence of sodium perchlorate may be corroborated by the weakest 457 cm^-1^ signature, which may be masked in the broad feature at 487 cm^-1^. These frequencies were observed in a previous study of NaClO_4_ [37]. Furthermore, in previous experimental and theoretical studies of Mars, a similar frequency was determined in perchlorate brines between 900–1000 cm^-1^ [27], and in a regolith model, a similar signature was found at 940 cm^-1^. In that case, it corresponds to the asymmetric mode υ(O-Cl-O) [28].

**Fig 5:**
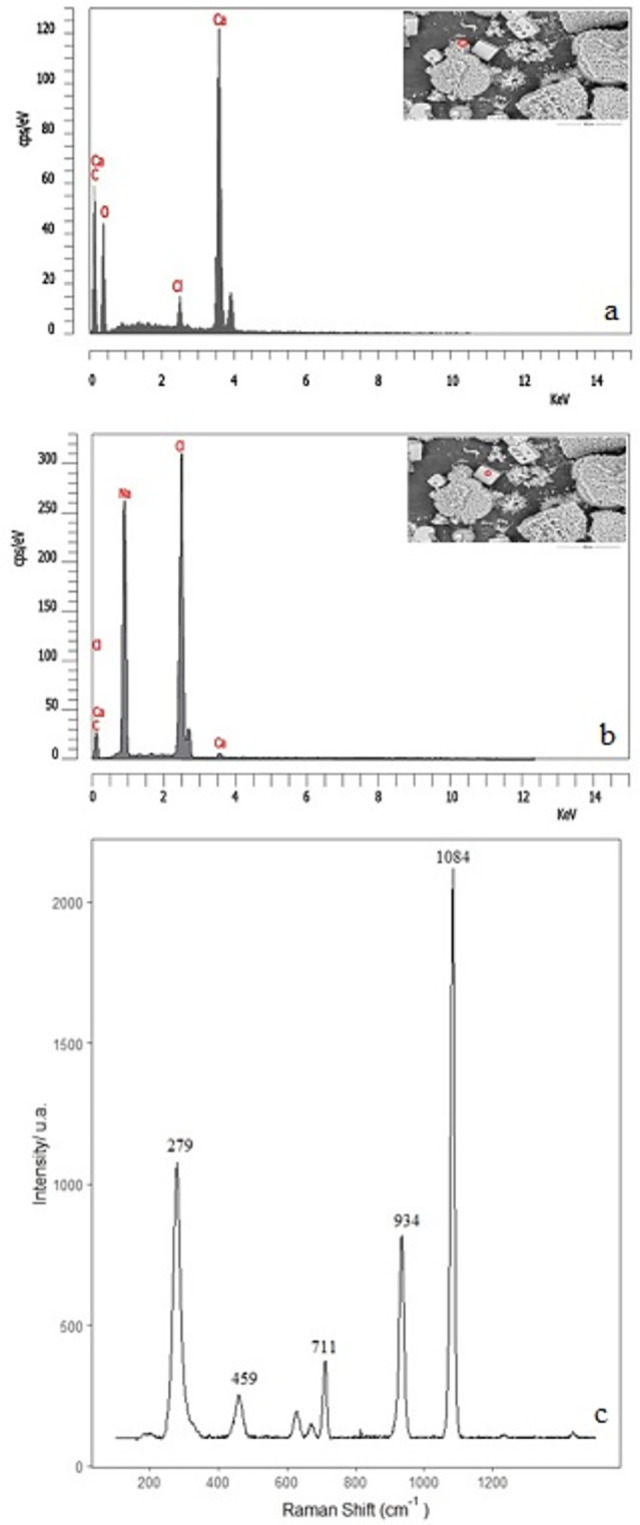
Chemical microanalysis of the internal surface of tubular structures formed from CaCl_2_ pellet sample with sodium perchlorate 2M dissolution (experiment C), possible formation of CaCO_3_. Micro-Raman spectra (at the bottom).

In the case of the precipitates obtained from pellet and seeds with perchlorate 2M dissolution (experiment C), the EDX analysis suggests that the flower’s petals are composed of CaCO_3_-like structures above (Fig 5A). On the contrary, the bright cubic crystals are NaCl (Fig 5B). The spectra have characteristic bands of calcite polymorphs (Fig 5C) at 280, 711 cm^-1^ and 1084, 705 cm^-1^ (within the 711 cm^-1^ broad peak) that correspond to calcite and aragonite [34–36]. In the same way, as in the case above, the υ(O-Cl-O) signal appears at 934 cm^-1^.

**Fig 6:**
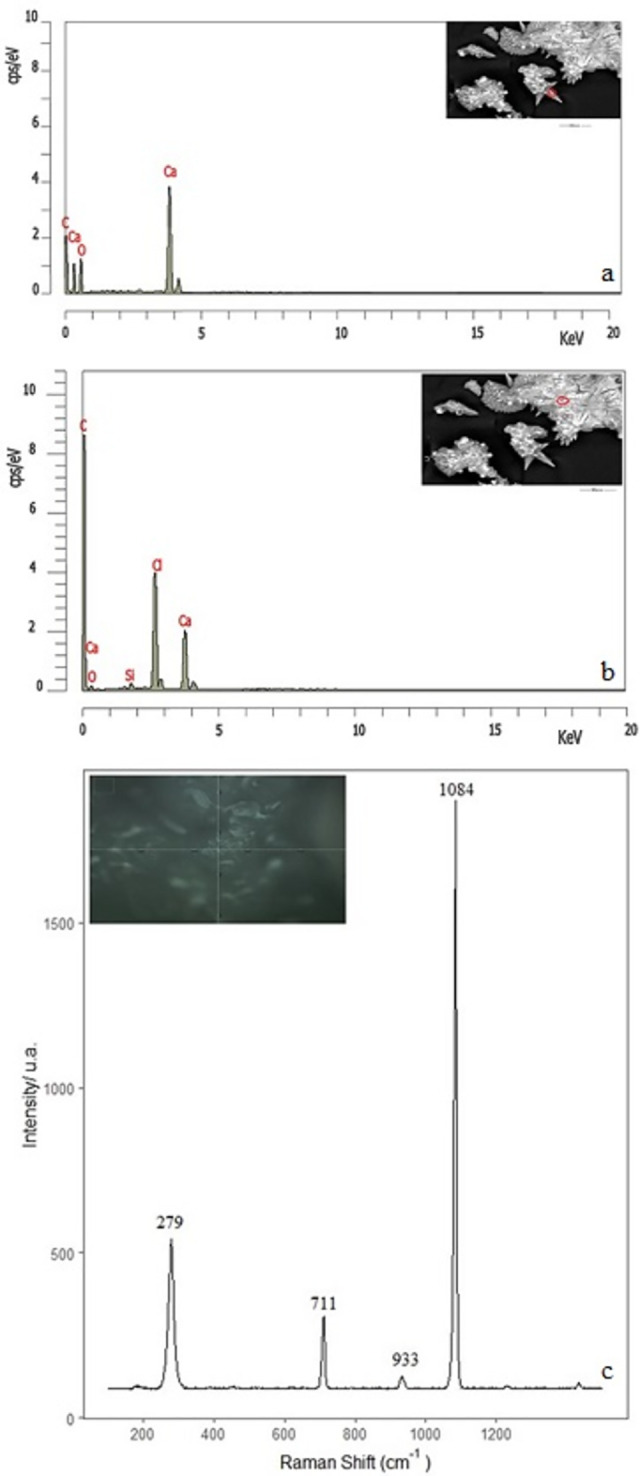
Chemical microanalysis of the internal surface of the sample formed from CaCl_2_ and sodium perchlorate dissolution 2M (experiment A), possible formation of CaCO_3_ in micro Raman with photo at 20x (bottom).

The needles aggregate and star-like structure were also investigated with EDX to determine the composition. The analysis indicates that in the star structure, the composition could correspond to CaCO_3_ (Fig 6A), whereas in the amorphous, it corresponds to CaCl_2_ (Fig 6B). This microphotograph corresponds to the sample formed with NaClO_4_ 2M (experiment A). The Raman analysis of petals (Fig 6C flowers image inside of the spectrum) shows the characteristic bands of the calcite polymorph at 1084, 711, and 280 cm^-1^ [36]. This result agrees with Oral and Ercan’s work, which proposes that flower morphology results from the re-growth processes of calcite particles [31].

**Fig 7:**
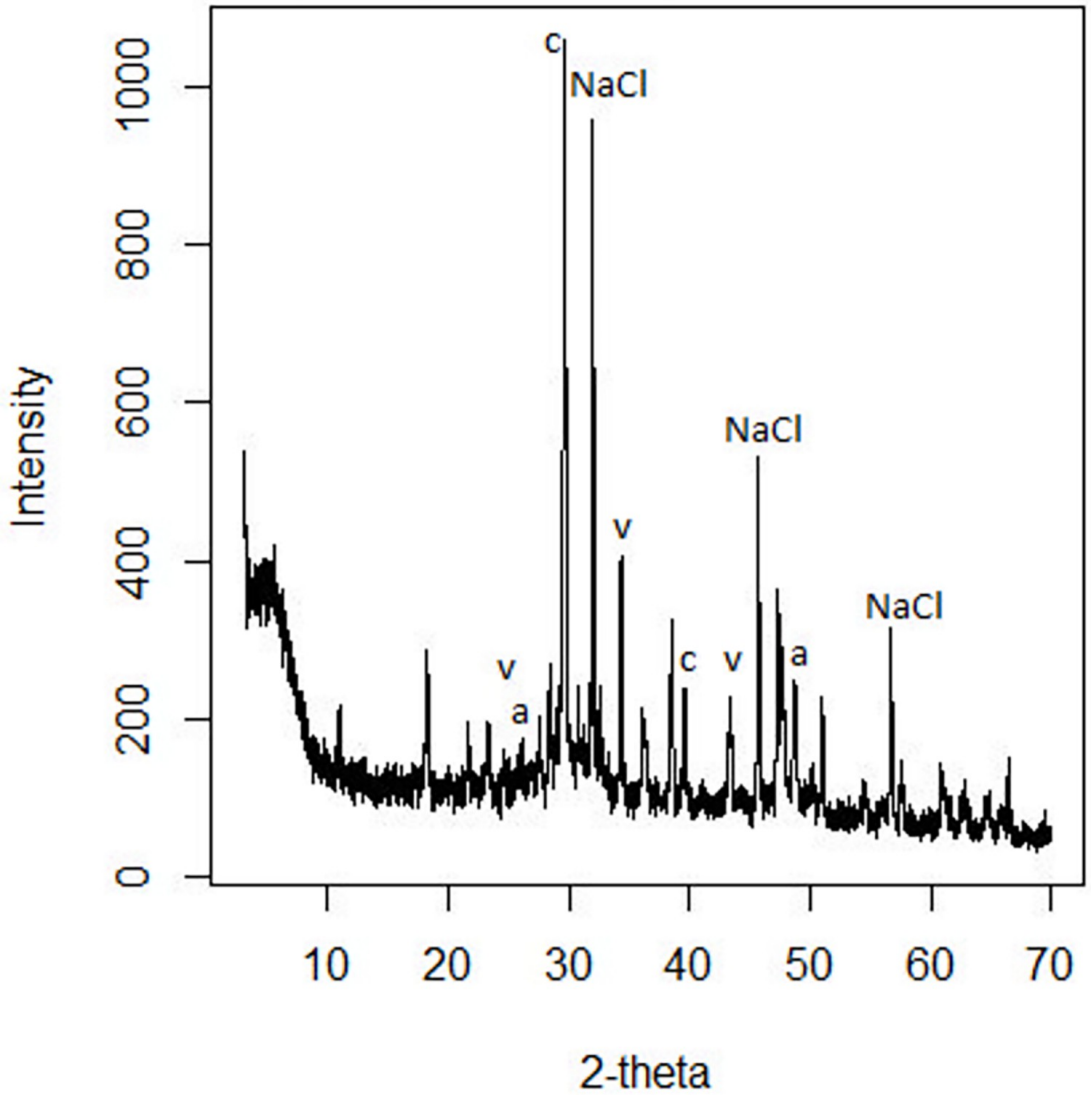
XPOWDER© x-ray diffraction patterns of the tubular structure obtained with CaCl_2_ and sodium perchlorate in terrestrial conditions. Reflections are assigned to polymorphs of calcite (c), vaterite (v) and aragonite (a).

The coexistence of calcium carbonate polymorphs can also be confirmed with the XRD pattern (Fig 7). The diffraction pattern shows the characteristic peaks with the following reflections: calcite at 29°, 39°, aragonite at 26°, 48° and vaterite at 24.8°, 33°, and 43° (2θ units). Other reflections at 27°, 32°, 45.5° and 57° correspond to NaCl salt. These values agree with previous studies of calcium carbonate formation [31, 32, 38, 39].

#### 2.2 Crystal growth of self-assembling structures with sulphate salts

**Fig 8:**
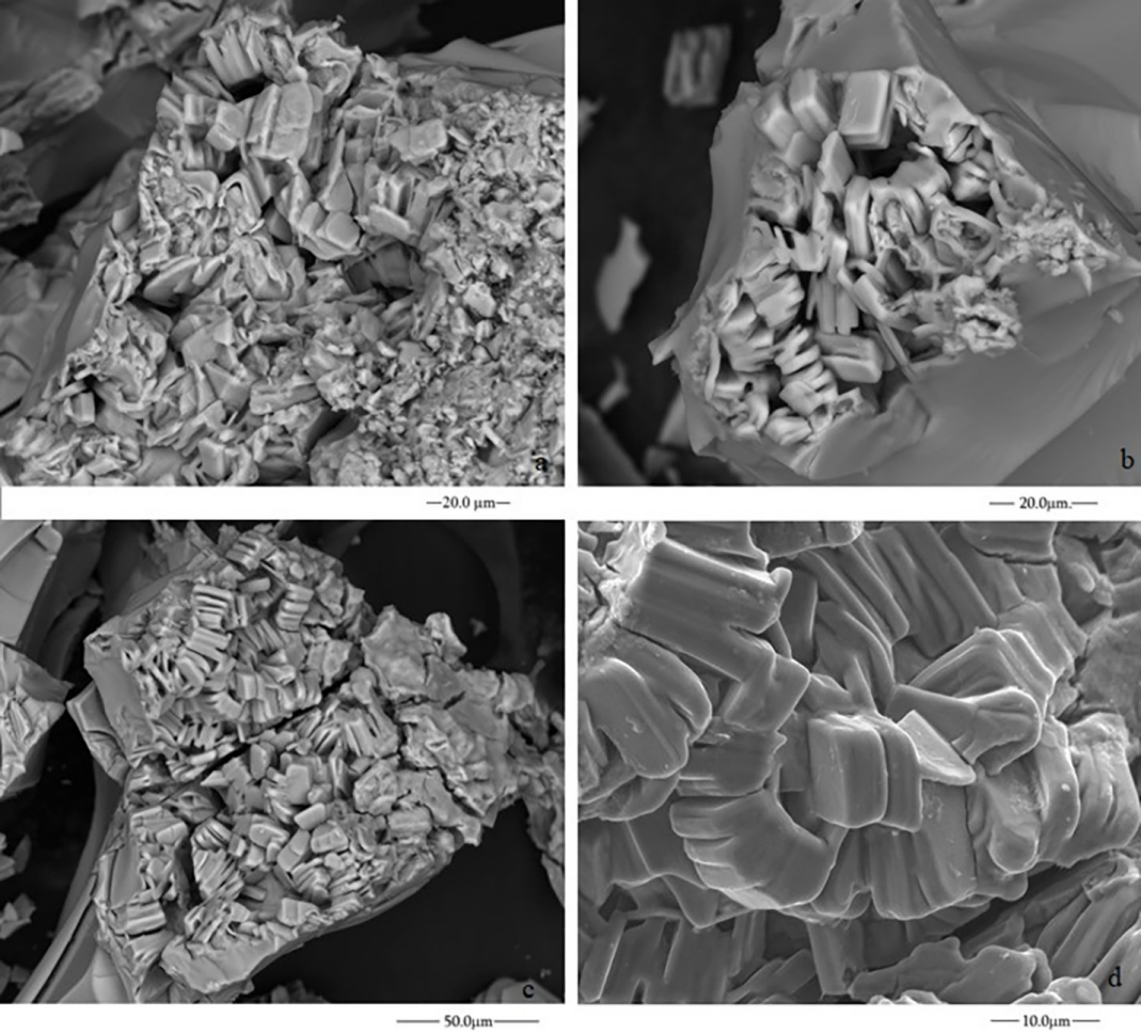
ESEM micrographs of the inner surface of the materials formed with FeSO_4_ and the potassium chlorate and sodium silicate dissolution (experiment E).

**Fig 9:**
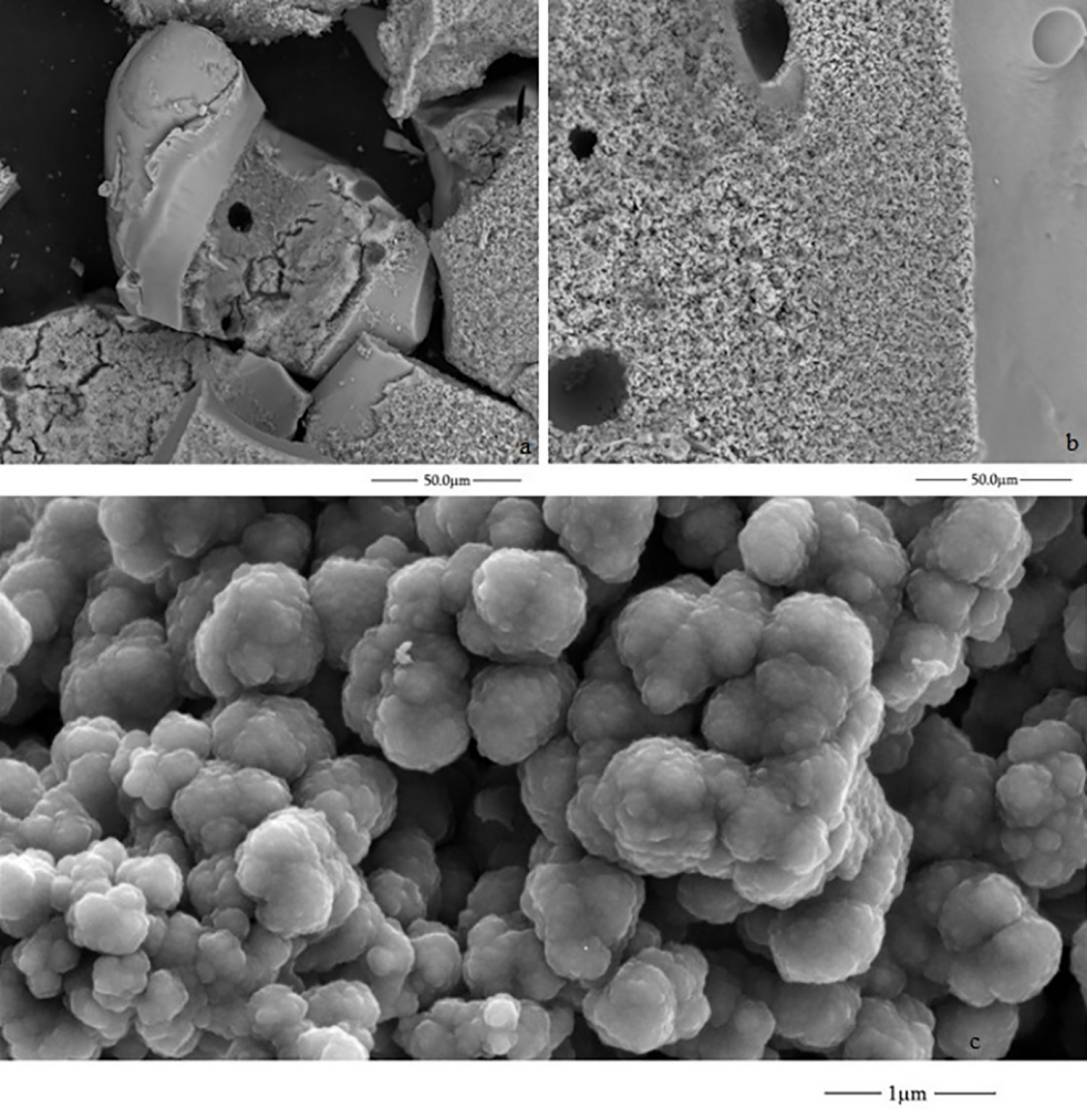
ESEM micrographs of the inner surface of the material formed with NiSO_4_ sodium perchlorate 2M (experiment C).

The self-assembling structures formed in experiments C and E were the only ones characterised by ESEM micrographs. They correspond to the experiments with NiSO_4_.7H_2_O seeds, sodium perchlorate 2M (experiment C) and FeSO_4_.7H_2_O seeds, and potassium chlorate and sodium silicate mixture dissolution (experiment E). In the case of iron sulphate, the crystals on the inner surface of the structure have rectangular shapes (Fig 8), while nickel sulphate presents a porous structure (Fig 9). The fine grains of Fig 9C are similar to those in ESEM microphotography of the characterisation of NiO nanoparticles [40].

**Fig 10:**
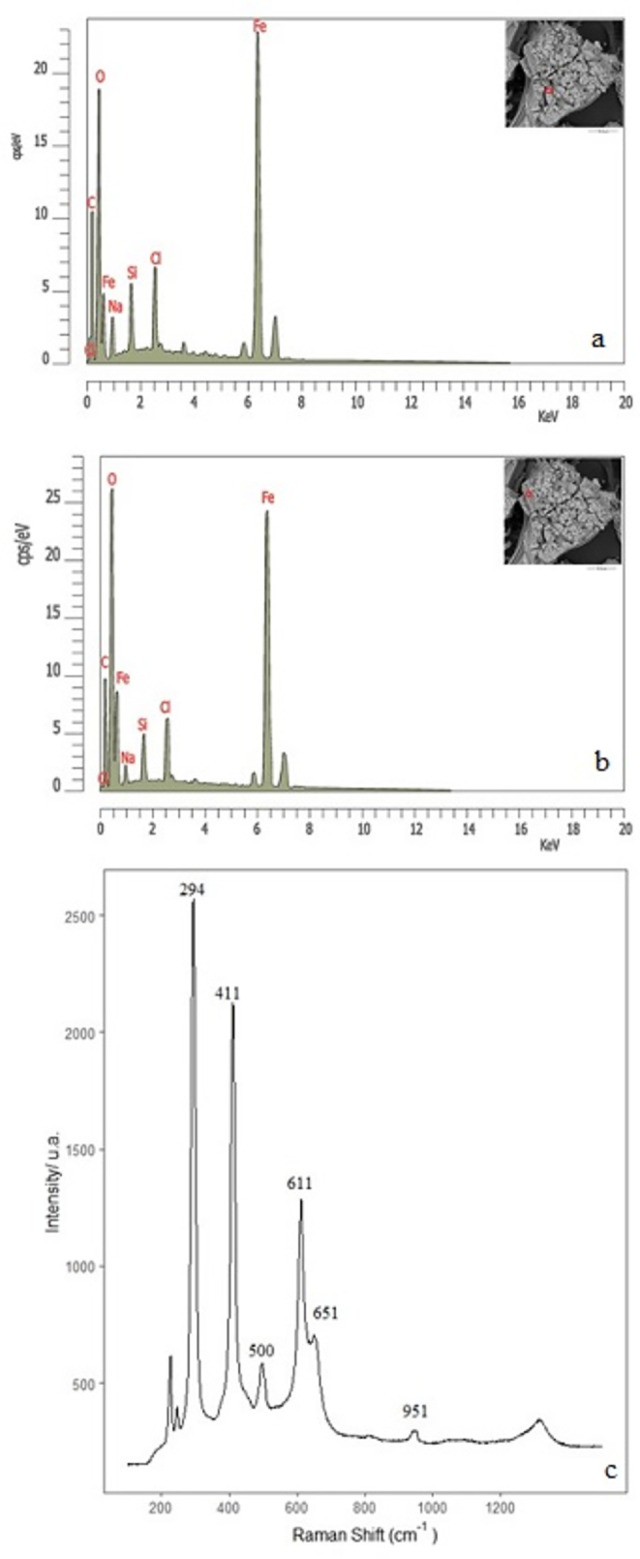
Chemical microanalysis of the internal surface of structures formed from FeSO_4_ and a mixture of potassium chlorate and sodium silicate dissolution (a and b). Raman spectrum (c) (experiment E).

For the case of precipitates formed in a sodium silicate and a potassium chlorate mixture, the EDX microanalysis suggests that the rectangular crystals could be formed of Fe, C, O, Cl and Si (Fig 10A and 10B). We have also performed a micro-Raman analysis to corroborate that the interior surface is rich in FeO/FeOH and determine a possible carbonatation process. The spectra show that the most intense peaks are characteristic of carbonate minerals such as siderite (FeCO_3_), whose peaks are found at 300 and 250 cm^-1^ and could be included in the broad peak at 294 cm^-1^. On the other hand, two peaks at 374 and 540 cm^-1^, including the broad feature at 411 and 500 cm^-1^, could be attributed tentatively to FeO/Fe(OH)_2_ found in goethite [24, 41]. This latter peak is in good agreement with previous work on tubular structures of iron sulfate [23, 24]. The characteristic peaks of sodium perchlorate structures appear at 951 and 457 cm^-1^ (the last one could be included in the widespread peak at 411 cm^-1^) (Fig 10C). Also, this frequency is close to the theoretical value of asymmetric mode υ(O-Cl-O) reported at 940 cm^-1^ [28]. The KClO_3,_ even in low concentration, has induced the formation of iron carbonate as a siderite mineral. This mineral was not found in our previous study [24], with sodium silicate and a dense CO_2_ atmosphere within the Space-Q Mars Chamber [42].

**Fig 11:**
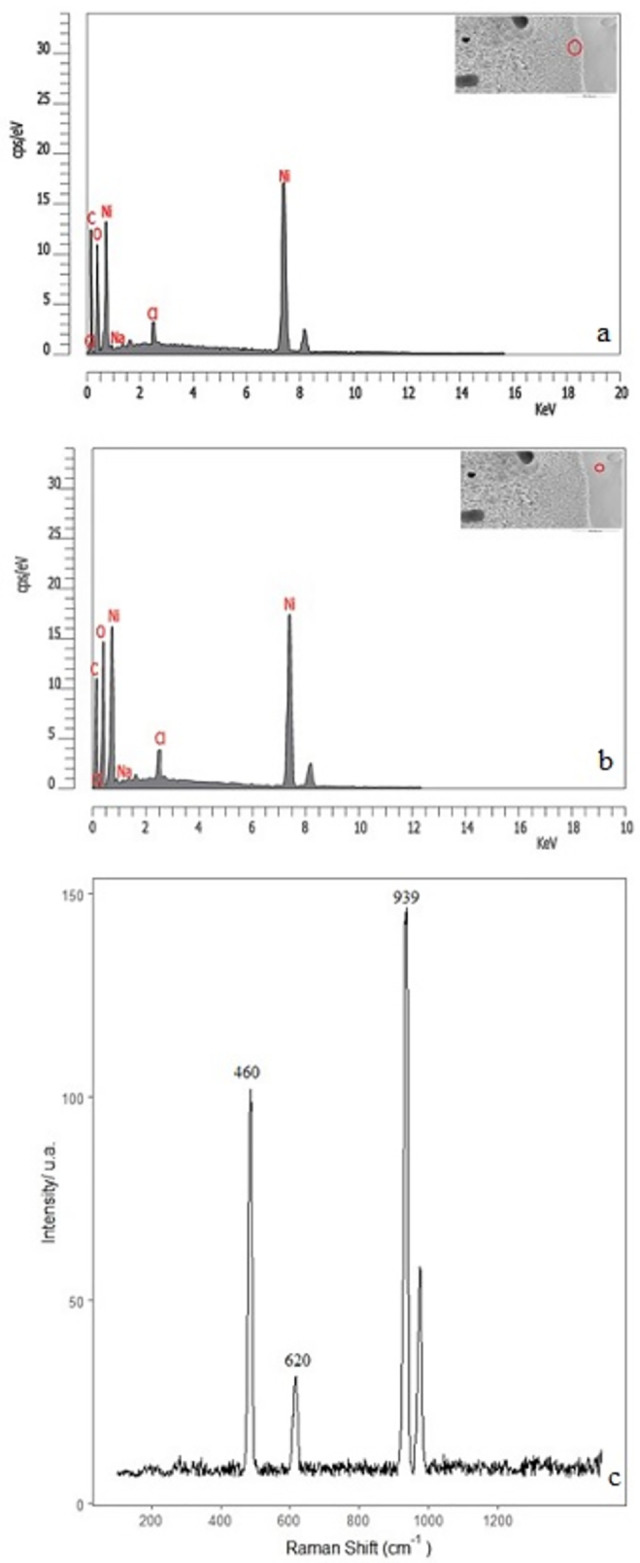
EDX microanalysis and Raman spectrum of the structures formed with NiSO_4_ in sodium perchlorate dissolution 2M (experiment C).

In addition, EDX analysis of the sample with NiSO_4_ (2M) shows a much higher proportion of oxygen, carbon, and nickel on the inside and external side of the structure, indicating the presence of carbonate and oxides-hydroxides Ni crystals that grow inside and outside of the structure (Fig 11A and 11B). This result differs from our previous results, in which the wall of the tubular structure was formed mainly by silicon, and the carbonates and oxy-hydroxy crystals grow in the inner of the material form [24–26].

**Fig 12:**
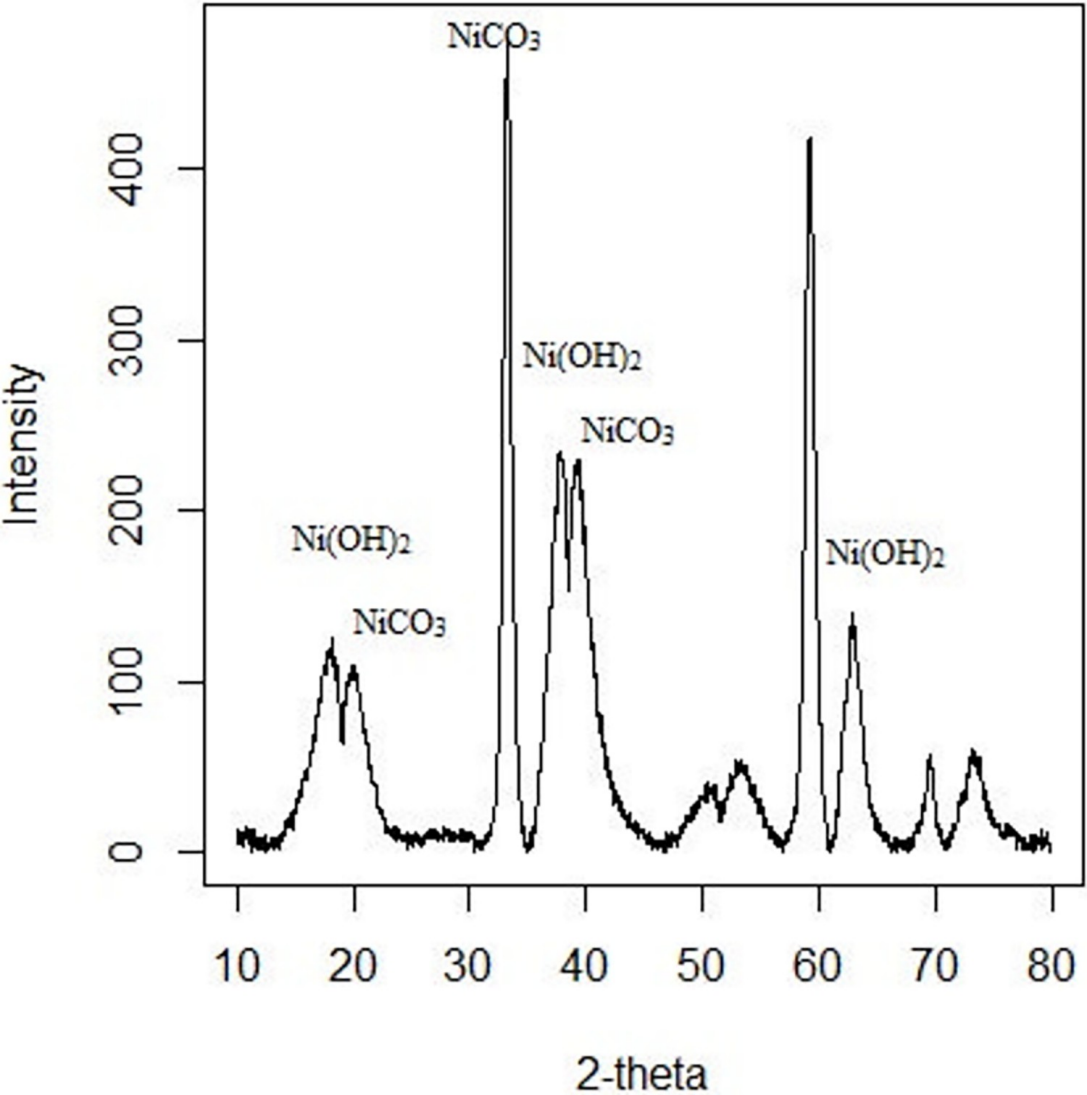
XPOWDER© x-ray diffraction patterns of the structure obtained with NiSO_4_ and sodium perchlorate. Reflections are assigned to NiCO_3_.

A Micro-Raman analysis corroborated that the surface’s interior and exterior are rich in nickel oxide/hydroxide and probably carbonates. The spectrum (Fig 11C) shows the characteristic bands at 939, 458 and 307 cm^-1^ that could correspond to Hellyerite mineral [Ni(CO_3_).6H_2_O], with peaks at 1050–1100, 930–940 and 300–460 cm^-1^ [41]. As discussed above in the iron sulphates sample, the peak that appears at 939 cm^-1^ also could correspond to υ(O-Cl-O). Other signals at 458 and 620 cm^-1^ can suggest the presence of nickel carbonate with sulphate, which may correspond to Burkeite mineral [Na_4_(SO_4_)(CO^3^)] whose characteristic signals appear at 450 and 630 cm^-1^ [24, 41]. On the other hand, the peaks in the theophrastite mineral (Ni(HO)_2_) appear at 300–310 and 445 cm^-1^ [41]. The later peak may perhaps be underneath the broad peak at 460 cm^-1^. We have not observed any signal of nickel oxide whose peaks appear at 200, 500 and 1090 cm^-1^ [40]. On the other hand, the DRX pattern of these samples (Fig 12) shows the characteristic diffraction peaks of the Ni hydroxylated phase at 19°, 39° and 63° (2θ units). These peaks are similar to those reported by Kotok [43]. Also, the signal of nickel carbonate was found at 20°, 30° and 40° (2θ units). These values were reported in the low crystallinity phases of the zaratite mineral [44]. Concerning the formation of this mineral (NiCO_3_), previous studies suggest that this must have been produced at a pH of 12 [45]. Our experimental setup reproduces similar structures and morphologies.

## Conclusions

The purpose of this work is to demonstrate the formation of carbonates through various geochemical reaction-precipitation processes under different simulated Martian conditions. We have conducted experiments using different aqueous solutions and solid grains from olivine and other volcanic or sedimentary rocks and minerals to illustrate a few examples of brine reaction-precipitation chemical paths that may have existed at the Jezero crater and elsewhere on Mars.

Similar experiments should be performed in the future under different environmental simulated Martian conditions (*i*.*e*. different CO_2_ pressures and rock support, using solid grains from olivine and other volcanic or sedimentary rocks and minerals that emulate those found at Jezero) for two main reasons: (i) to demonstrate the microscopic scale analysis and validate the procedures and instrumentation of the mars sample receiving facility in preparation for the analysis of carbonates produced in natural brine-rock carbonation processes and, (ii) to prepare synthetic carbonates that may serve as control experiments for any future analysis about the carbon composition and distribution of the Mars Sample Return collection.

Our experiments have shown that calcium chloride and sulphate salts can quickly form carbonate structures with sodium perchlorate solution under Martian-like conditions. The ESEM’s micrographs and chemical microanalysis suggest that brines of sulphates (Fe and Ni) and CaCl_2_ salts with sodium perchlorate and mixed solutions of sodium silicate and potassium chlorate in contact with an atmosphere with merely 400 ppb of CO_2_ can fix atmospheric CO_2_, forming microstructures of calcium carbonate with various biomorphic morphologies. The observed micro-scale morphologies are assigned to different calcium carbonate polymorphs such as calcite, vaterite, and aragonite. Other carbonates, such as siderite and Ni(CO_3_), are also formed. Additionally, the precipitates from sulphate can produce other species that can appear on the inner side of the structures, such as FeO, Fe(OH)_2_ and Ni(OH)_2_, as confirmed by Micro-Raman spectroscopy and X-ray diffraction.

Our analysis suggests that carbonates such as calcium carbonate and their polymorphs, nickel carbonate and siderite, may form from the precipitation of sodium perchlorate brines interacting with other salts. Different types of precipitates formed only under very high pH conditions and distributed sparsely within the sample, such as nickel carbonate, suggest that the pH can vary enormously even within a small reaction area of only a few centimetres. Therefore, some caution is needed to interpret the aqueous history sequence of an environment, mainly when this is supported only by detecting a variety of mixed minerals that may form from precipitation under different pH conditions. These experiments show that all the precipitates may have been produced simultaneously -in a “one-pot” reaction- and still be exposed to different micro-scale pH conditions.

It is important to note that while carbonates can form biomorphs visible at the microscopic scale, they should not be misinterpreted as valid biomarkers, as they can be produced abiotically. The experimental study of the carbonate precipitates that are produced in the reactions of minerals with brines and CO_2_ may be helpful to interpret the paleo-precipitation conditions that existed once on Mars, to distinguish abiotic from biological processes, discriminate biomorphs, and to prepare analogue materials for laboratory analysis in preparation for the analysis of the Mars sample return collection. Similar experiments may also be used to quantify how much CO_2_ may have been sequestered from the atmosphere on Mars throughout the history of the evolution of Mars.

## Supporting information

S1 File (DOCX)

S2 File (PDF)

S3 File (ZIP)

## References

[1] Scheller EL, Hollis JR, Cardarelli EL, Steele A, Beegle LW, et al. (2022) Aqueous alteration processes in Jezero crater, Mars: implications for organic geochemistry. Science 378: eabo5204. doi: 10.1126/science.abo5204 36417498

[2] Farley KA, Stack KM, Shuster DL, Horgan BHN, Hurowitz JA, et al. (2022) Aqueously altered igneous rocks sampled on the floor of Jezero crater, Mars. Science 377: eabo2196. doi: 10.1126/science.abo2196 36007009

[3] Liu Y, Tice MM, Schmidt ME, Treiman AH, Kizovski TV, et al. (2022) An olivine cumulate outcrop on the floor of Jezero crater, Mars. Science 377: 1513–1519. doi: 10.1126/science.abo2756 36007094

[4] Meyer MA, Kminek G, Beaty DW, Carrier BL, Haltigin T, et al. (2022) Final Report of the Mars Sample Return Science Planning Group 2 (MSPG2). Astrobiology 22: S5–S26. doi: 10.1089/AST.2021.0121 34904888

[5] Tice MM, Hurowitz JA, Allwood AC, Jones MWM, Orenstein BJ, et al. (2022) Alteration history of Séítah formation rocks inferred by PIXL x-ray fluorescence, x-ray diffraction, and multispectral imaging on Mars. Science Advances 8: eabp9084. 36417516 10.1126/sciadv.abp9084 PMC9683721

[6] Ehlmann BL, Mustard JF, Murchie SL, Poulet F, Bishop JL, et al. (2008) Orbital Identification of Carbonate-Bearing Rocks on Mars. Science 322: 1828–1832. doi: 10.1126/science.1164759 19095939

[7] Goudge TA, Mustard JF, Head JW, Fassett CI, Wiseman SM (2015) Assessing the mineralogy of the watershed and fan deposits of the Jezero crater paleolake system, Mars. Journal of Geophysical Research: Planets 120: 775–808.

[8] Horgan BHN, Anderson RB, Dromart G, Amador ES, Rice MS (2020) The mineral diversity of Jezero crater: Evidence for possible lacustrine carbonates on Mars. Icarus 339: 113526.

[9] Brown AJ, Viviano CE, Goudge TA (2020) Olivine‐carbonate mineralogy of the Jezero crater region. Journal of Geophysical Research: Planets 125: e2019JE006011. doi: 10.1029/2019je006011 33123452 PMC7592698

[10] Zastrow AM, Glotch TD (2021) Distinct Carbonate Lithologies in Jezero Crater, Mars. Geophysical Research Letters 48: e2020GL092365. doi: 10.1029/2020GL092365 34219844 PMC8243932

[11] Bosak T, Moore KR, Gong J, Grotzinger JP (2021) Searching for biosignatures in sedimentary rocks from early Earth and Mars. Nature Reviews Earth & Environment 2: 490–506.

[12] Sharma S, Roppel RD, Murphy AE, Beegle LW, Bhartia R, et al. (2023) Diverse organic-mineral associations in Jezero crater, Mars. Nature 619: 724–732. doi: 10.1038/s41586-023-06143-z 37438522 PMC10371864

[13] McKay DS, Gibson EK, Thomas-Keprta KL, Vali H, Romanek CS, et al. (1996) Search for Past Life on Mars: Possible Relic Biogenic Activity in Martian Meteorite ALH84001. Science 273: 924–930. doi: 10.1126/science.273.5277.924 8688069

[14] Perri E, Tucker ME, Spadafora A (2012) Carbonate organo-mineral micro- and ultrastructures in sub-fossil stromatolites: Marion lake, South Australia. Geobiology 10: 105–117. 22039973 10.1111/j.1472-4669.2011.00304.x

[15] Gázquez F, Rull F, Calaforra JM, Venegas G, Manrique JA, et al. (2014) Mineralogical and geochemical characterization of hydrated minerals from subterranean environments: implications for planetary exploration. Estudios Geológicos 70.

[16] Halevy I, Fischer WW, Eiler JM (2011) Carbonates in the Martian meteorite Allan Hills 84001 formed at 18 ± 4°C in a near-surface aqueous environment. Proceedings of the National Academy of Sciences 108: 16895–16899. 10.1073/pnas.1109444108 PMC3193235 21969543

[17] Niles PB, Catling DC, Berger G, Chassefière E, Ehlmann BL, et al. (2013) Geochemistry of Carbonates on Mars: Implications for Climate History and Nature of Aqueous Environments. 174: 301–328.

[18] Gendrin A, Mangold N, Bibring J-P, Langevin Y, Gondet B, et al. (2005) Sulfates in Martian Layered Terrains: The OMEGA/Mars Express View. Science 307: 1587–1591. doi: 10.1126/science.1109087 15718429

[19] Bristow TF, Grotzinger JP, Rampe EB, Cuadros J, Chipera SJ, et al. (2021) Brine-driven destruction of clay minerals in Gale crater, Mars. Science 373: 198–204. doi: 10.1126/science.abg5449 34244410

[20] Bandfield JL, Glotch TD, Christensen PR (2003) Spectroscopic Identification of Carbonate Minerals in the Martian Dust. Science 301: 1084–1087. doi: 10.1126/science.1088054 12934004

[21] Cartwright JH, Escribano B, Sainz-Díaz CI (2011) Chemical-garden formation, morphology, and composition. I. Effect of the nature of the cations. Langmuir 27: 3286–3293. doi: 10.1021/la104192y 21391635

[22] Barge LM, Cardoso SSS, Cartwright JHE, Cooper GJT, Cronin L, et al. (2015) From Chemical Gardens to Chemobrionics. Chemical Reviews 115: 8652–8703. doi: 10.1021/acs.chemrev.5b00014 26176351

[23] Barge LM, Cardoso SSS, Cartwright JHE, Doloboff IJ, Flores E, et al. (2016) Self-assembling iron oxyhydroxide/oxide tubular structures: laboratory-grown and field examples from Rio Tinto. Proceedings of the Royal Society A: Mathematical, Physical and Engineering Science 472. doi: 10.1098/rspa.2016.0466 27956875 PMC5134306

[24] Escamilla-Roa E, Zorzano M-P, Martín-Torres FJ, Sainz-Díaz C, Cartwright J (2022) Self-Assembled Structures Formed in CO_2_ -Enriched Atmospheres: A Case-Study for Martian Biomimetic Forms. Astrobiology 22: 863–879. doi: 10.1089/ast.2021.0123 35613388

[25] Escamilla-Roa E, Cartwright JHE, Sainz-Díaz CI (2019) Chemobrionic Fabrication of Hierarchical Self-Assembling Nanostructures of Copper Oxide and Hydroxide. ChemSystemsChem 1: e1900011.

[26] Sainz-Díaz CI, Escamilla-Roa E, Cartwright JHE (2018) Growth of Self-Assembling Tubular Structures of Magnesium Oxy/Hydroxide and Silicate Related With Seafloor Hydrothermal Systems Driven by Serpentinization. Geochemistry, Geophysics, Geosystems 19: 2813–2822.

[27] Nikolakakos G, Whiteway JA (2018) Laboratory study of adsorption and deliquescence on the surface of Mars. Icarus 308: 221–229.

[28] Escamilla-Roa E, Zorzano M-P, Martin-Torres J, Hernández-Laguna A, Ignacio Sainz-Díaz C (2020) DFT study of the reduction reaction of calcium perchlorate on olivine surface: Implications to formation of Martian’s regolith. Applied Surface Science 512: 145634.

[29] Martin JD (2004) XPowder software.

[30] Reddy MM, Nancollas GH (1976) The crystallization of calcium carbonate: IV. The effect of magnesium, strontium and sulfate ions. Journal of Crystal Growth 35: 33–38.

[31] Oral ÇM, Ercan B (2018) Influence of pH on morphology, size and polymorph of room temperature synthesized calcium carbonate particles. Powder Technology 339: 781–788.

[32] Yang L-f Chu D-q, Sun H-l, Ge G(2016) Room temperature synthesis of flower-like CaCO3 architectures. New Journal of Chemistry 40: 571–577.

[33] Rojas Cáceres JP, Yazdani-Pedram Zobeiri M(2008) Estudio mineralización de carbonato de calcio usando como matriz quitosano y mezclas de quitosano con polímeros sintéticos hidrosolubles. Santiago: Memoria (químico)—Universidad de Chile, 2008.

[34] Cardoso SSS, Cartwright JHE, Checa AG, Sainz-Díaz CI (2016) Fluid-flow-templated self-assembly of calcium carbonate tubes in the laboratory and in biomineralization: The tubules of the watering-pot shells, Clavagelloidea. Acta Biomaterialia 43: 338–347. 27402180 10.1016/j.actbio.2016.07.005

[35] Wehrmeister U, Jacob DE, Soldati AL, Loges N, Häger T, et al. (2011) Amorphous, nanocrystalline and crystalline calcium carbonates in biological materials. Journal of Raman Spectroscopy 42: 926–935.

[36] Kontoyannis CG, Vagenas NV (2000) Calcium carbonate phase analysis using XRD and FT-Raman spectroscopy. Analyst 125: 251–255.

[37] Miller AG, Macklin JW (1985) Vibrational spectroscopic studies of sodium perchlorate contact ion pair formation in aqueous solution. The Journal of Physical Chemistry 89: 1193–1201.

[38] Takabait F, Mahtout L, Pérez Villarejo L, Carrasco Hurtado B, Sánchez Soto PJ (2016) Obtención de nanopartículas de carbonato de calcio a partir de precursores inorgánicos y sacarosa como aditivo con potencial utilización como biomaterial. Boletín de la Sociedad Española de Cerámica y Vidrio 55: 179–184.

[39] Kim B-J, Park E-H, Choi K, Kang K-S (2017) Synthesis of CaCO_3_ using CO_2_ at room temperature and ambient pressure. Materials Letters 190: 45–47.

[40] Baghbanian SM (2014) Synthesis, characterization, and application of Cu_2_O and NiO nanoparticles supported on natural nanozeolite clinoptilolite as a heterogeneous catalyst for the synthesis of pyrano[3,2-b]pyrans and pyrano[3,2-c]pyridones. RSC Advances 4: 59397–59404.

[41] Lafuente B, Downs RT, Yang H, Stone N (2015) The power of databases: The RRUFF project. Berlin: W. De Gruyter.

[42] Vakkada Ramachandran A, Nazarious MI, Mathanlal T, Zorzano M-P, Martín-Torres J (2020) Space Environmental Chamber for Planetary Studies. Sensors 20: 3996. doi: 10.3390/s20143996 32708384 PMC7412122

[43] Kotok V, Кovalenko V (2017) Optimization of nickel hydroxide electrode of the hybrid supercapacitor. Eastern-European Journal of Enterprise Technologies 1: 4–9.

[44] La Iglesia A, Garcia-Guinea J, González del Tánago J (2014) La zaratita de Cabo Ortegal (A Coruña): historia de su descubrimiento y caracterización actual. Estudios Geológicos 70: e003.

[45] Mallya RM, Murthy ARV (1961) Studies on the basic carbonates of nickel part VII: Formation and configurations of basic nickel carbonates Journal of the Ïndian Institute Science 43.

