# Supplementary material for: Experimental formation of carbonates from perchlorate and sulphate brines: Implications for Jezero crater, Mars
Source: PLoS One. 2024 Dec 5;19(12):e0312495. doi: 10.1371/journal.pone.0312495 (PMC11620553; doi:10.1371/journal.pone.0312495)
Supplement: S1 File — (DOCX) [file pone.0312495.s001.docx]

Attn. PLOS ONE staff

Burgos, October 16, 2024

Dear PLOS ONE staff

I send you enclosed a revision of the manuscript Ref: PONE-D-24-14664R1entitled "Experimental formation of carbonates from perchlorate and sulphate brines: implications for Jezero crater, Mars " by Elizabeth Escamilla-Roa, Javier Martin-Torres and Maria-Paz Zorzano. We have updated the article considering comments provided by PLOS ONE staff. We include the manuscript incorporating the required changes related with the figures (JOURNAL REQUIREMENTS), the minor modifications in this version are as marked-up manuscript for better tracking of modifications. We have also included the JOURNAL REQUIREMENTS letter responding to each point. We hope that this new version will be suitable for publication.

JOURNAL REQUIREMENTS:

1. We note that you have uploaded figures in multiple parts (i.e. Fig 1A, 1B, etc.). Please be advised that PLOS ONE does not publish figures in multiple parts. To ensure that your figures publish correctly, we request that you either separate the multiple parts out into their own figures (i.e. Fig 1A becomes Fig 1, 1B becomes Fig 2, etc.) or that all parts are consolidated into one single figure file, and reupload the files. You can refer to our guidelines for multipanel

figure files here: https://journals.plos.org/plosone/s/figures#loc‐multi‐panel‐figures.

**“Author reply”**:

- In the manuscript, we have changed the word Figure to Fig and they are shown with the change control and in yellow color.

- Figures have been corrected according to PLOS ONE format and have been uploaded.

- Please verify that the author contributions have been changed for Javier and Mari Paz as follows:

Javier Martin-Torres:

- Conceptualization

- Supervision

- Writing – review & editing

María-Paz Zorzano:

- Funding acquisition

- Supervision

- Writing – original draft

- Investigation

- Writing – review & editing

Thank you for your attention

**Elizabeth Escamilla Roa**

Molecular Simulation

[ICCRAM – Universidad de Burgos](https://www.ubu.es/iccram)

e-mail: eescamilla@ubu.es
