# Supplementary material for: Experimental formation of carbonates from perchlorate and sulphate brines: Implications for Jezero crater, Mars
Source: PLoS One. 2024 Dec 5;19(12):e0312495. doi: 10.1371/journal.pone.0312495 (PMC11620553; doi:10.1371/journal.pone.0312495)
Supplement: S2 File — (PDF) [file pone.0312495.s002.pdf]

Attn. PLOS ONE staff

Burgos, October 12, 2024

Dear PLOS ONE staff

I send you enclosed a revision of the manuscript Ref: PONE-D-24-14664R1_FTC entitled "Experimental formation of carbonates from perchlorate and sulphate brines: implications for Jezero crater, Mars " by Elizabeth Escamilla-Roa, Javier Martin-Torres and Maria-Paz Zorzano. We have updated the article considering comments provided by PLOS ONE staff. We include the manuscript incorporating the required changes (JOURNAL REQUIREMENTS) and also, include minor modifications in the text as a marked-up manuscript for better tracking of modifications. We have also included the JOURNAL REQUIREMENTS letter responding to each point. We hope that this new version will be suitable for publication.

JOURNAL REQUIREMENTS:

1. Please ensure that the author list and affiliations are correct on the title page of your manuscript, and that your author contributions, competing interests, and financial dis closure are correct as listed below. All of these sections will be indexed in PubMed an d published by PLOS ONE as you have written them. Please email plosone@plos.org if any changes to this content need to be made. Elizabeth Escamilla-Roa: Conceptualization Data curation 2
Formal analysis Investigation Writing – original draft Javier Martin-Torres: Supervision Writing – review & editing María-Paz Zorzano: Funding acquisition Supervision Writing – review & editing

“Author reply”:

- We have changed some words in the manuscript and they are shown with the change control in yellow color.

- We have changed the affiliations in the manuscript as following:

Elizabeth Escamilla-Roa,[1,#a]

[1] Instituto Andaluz de Ciencias de la Tierra (CSIC-UGR), 18100, Av. de las Palmeras 4, Armilla, Granada, Spain.

[#a] Current Address: International Research Center in Critical Raw Materials for Advanced Industrial Technologies (ICCRAM), University of Burgos, 09001, Burgos, Spain

María-Paz Zorzano[3]

[3]Centro de Astrobiología (CAB), CSIC-INTA, 28850, Torrejón de Ardoz, Madrid, Spain

-We want to change the author contributions for Javier and Mari Paz as follows:

Javier Martin-Torres:
- Conceptualization
- Supervision
- Writing – review & editing

María-Paz Zorzano:
- Funding acquisition
- Supervision
- Writing – original draft
- Investigation
- Writing – review & editing

Please ensure that the Competing Interests and Financial Disclosure statements listed below ar e suitable for publication. These sections will be indexed in PubMed and published by PLOS O NE as you have written them. Please email plosone@plos.org if any changes to these stateme nts need to be made. Competing Interests: The authors have declared that no competing inte rests exist. Financial Disclosure: M.-P.Z. was supported by grant PID2022-140180OB-C21 funded by MCIU/AEI /10.13039/501100011033 / FEDER, UE. European COST Action CA17 120 Spanish MINECO project FIS2016-77692-C2-2P. The funders had no role in study design, data collection and analysis, decision to publish, o r preparation of the manuscript.

"Author reply":

Competing Interests and Financial Disclosure statements are correct

2. We note that figure/s [5-12] have not been uploaded as separate files. Please ensure that all figures are upload ed to the submission system as a separate file in the file inventory.

"Author reply":

The remaining figures have been uploaded

3. To prevent production delays, we recommend using the Author Formatting Checklist to confirm that your paper meets PLOS ONE's typesetting requirements for

References, Tables, and Figures:http://journals.plos.org/plosone/s/file?id=c819/plos-one-author-formatting-checklist.docx. This checklist is a reference tool for you; please do not upload the completed Author Formatting Checklist with your submission files.

“Author reply”:

Done

4. To ensure your figures meet our technical requirements, please run each figure included in your submission files through the PACE tool: https://pacev2.apexcovantage.com/. PACE will assess whether your figures meet our technical requirements and will fix the figure(s) or identify any problem(s) that cannot be automatically fixed. It can also convert figures to TIFF format, resize, and rename figures to meet our naming conventions. To use PACE, first register as a user. Follow the instructions on the site for assessing and converting your figure files. If you experience any difficulty using this tool or have questions about any of the figures and/or images in your paper, please inform the journal office in your response letter.

“Author reply”:

Done

Thank you for your attention

Elizabeth Escamilla Roa

Molecular Simulation

ICCRAM – Universidad de Burgos

e-mail: eescamilla@ubu.es
